# Detection of gastric cancer and its histological type based on iodine concentration in spectral CT

**DOI:** 10.1186/s40644-018-0176-2

**Published:** 2018-11-09

**Authors:** Rui Li, Jing Li, Xiaopeng Wang, Pan Liang, Jianbo Gao

**Affiliations:** 1 Department of Radiology, the First Affiliated Hospital of Zhengzhou University, No. 1, East Jianshe Road, Zhengzhou, 450052 Henan China; 2 Department of Radiology, the Affiliated Cancer Hospital of Zhengzhou University, Henan Cancer Hospital, No. 127, Dongming Road, Zhengzhou, 450008 Henan China

**Keywords:** Gastric, Adenocarcinoma, Spectral CT imaging, Iodine concentration, Histological degree

## Abstract

**Background:**

Computed tomography (CT) imaging is the most common imaging modality for the diagnosis and staging of gastric cancer. The aim of this study is was to prospectively explore the ability of quantitative spectral CT parameters in the detection of gastric cancer and its histologic types.

**Methods:**

A total of 87 gastric adenocarcinoma (43 poorly and 44 well-differentiated) patients and 36 patients with benign gastric wall lesions (25 inflammation and 11 normal), who underwent dual-phase enhanced spectral CT examination, were retrospectively enrolled in this study. Iodine concentration (IC) and normalized iodine concentration (nIC) during arterial phase (AP) and portal venous phase (PP) were measured thrice in each patient by two blinded radiologists. Moreover, intraclass correlation coefficient (ICC) was used to assess the interobserver reproducibility. Differences of IC and nIC values between gastric cancer and benign lesion groups were compared using Mann-Whitney U test. Furthermore, the gender, age, location, thickness and histological types of gastric adenocarcinoma were analyzed by Mann-Whitney U test or Kruskal-Wallis H test. Receiver operating characteristic (ROC) curves were used to evaluate the diagnostic efficacy of IC and nIC values, and the optimal cut-off value was calculated with Youden J.

**Results:**

An excellent interobserver agreement (ICC >  0.6) was achieved for IC. Notably, the values of ICAP, ICPP, nICAP and nICPP were significantly higher in gastric cancer group (*Z* = 5.870, 3.894, 2.009 and 10.137, respectively; *P* < 0.05) than those in benign lesion group. Additionally, the values of ICAP, ICPP, nICAP and nICPP were significantly higher in poorly differentiated gastric adenocarcinoma group (*Z* = 4.118, 5.637, 6.729 and 2.950, respectively; *P* < 0.005) than those in well-differentiated gastric adenocarcinoma group. There were no statistically significant differences in the values of ICAP, ICPP, nICAP and nICPP between age, gender, tumor thickness and tumor location. Furthermore, the area under the curve (AUC) values of ICAP, nICAP, ICPP and nICPP were 0.745, 0.584, 0.662, and 0.932, respectively, for gastric cancer detection; while 0.756, 0.919, 0.851 and 0.684, respectively, in discriminating poorly differentiated gastric adenocarcinoma.

**Conclusion:**

IC values exhibited great potential in the preoperative and non-invasive diagnosis of gastric cancer and its histological types. In particular, nICPP is more effective for the identification of gastric cancer, whereas nICAP is more effective in discriminating poorly differentiated gastric adenocarcinoma.

## Background

Gastric cancer is the fifth most common cancer and the third leading cause of cancer-related deaths worldwide [1]. Gastric adenocarcinoma comprises 95% of all gastric cancers [2]. The incidence and mortality rates associated with gastric adenocarcinoma are both the highest among all malignant tumors of the digestive tract in China [3, 4], representing an emerging threat to human health. The mean survival time of patients with advanced gastric cancer is less than 1 year [5]. Therefore, early detection, diagnosis and treatment are advocated to improve the clinical outcomes and quality of life in patients with gastric cancer.

Histological grading has been considered a predictor of lymph node metastasis and poor survival in gastric cancer [6]. Hence, accurate assessment of histological types is crucial for individualizing patient management [7]. Gold standard for the diagnosis of gastric cancer and its histological types can be obtained through preoperative endoscopic biopsy in clinical practice. However, endoscopic biopsy is an invasive procedure, and may posses unavoidable sampling bias and incoincident with histological diagnosis during surgery [8]. As compared to invasive endoscopic biopsy, preoperative imaging technique offers many advantages as its non-invasive detection and histologic evaluation of tumors, as well as the assessment of regional or distant lymph node metastasis. Conventional contrast-enhanced CT imaging is the first-line imaging modality for the detection and staging of gastric cancer. Its combination with multiple planar reconstruction and virtual endoscopy has proven to be effective for the diagnosis of gastric wall invasion in patients with gastric cancer [9]. However, this technique relies solely on the morphological criteria, and lack of parameters for quantitative analysis.

Spectral CT provides material decomposition (MD) images that can quantitatively map the iodine concentration (IC) in the enhanced images of tissues. This IC value has been found to be strongly correlated with the actual IC in the phantom [10]. Preliminary studies have reported the use of IC value in differentiating benign from malignant lesions, evaluating tumor, node, and metastasis (TNM) staging and determining the efficacy of anticancer therapy [11–16]. However, to the best of our knowledge, only a few studies have employed IC value in discriminating the histological types of gastric adenocarcinoma [12, 17], and the results are inconsistent with respect to arterial phase (AP) and portal venous phase (PP). Indeed, the application of IC values for the discrimination of gastric cancer and its histological types is still in the exploratory stage.

Therefore, this study aimed to evaluate the diagnostic efficacy of IC values for the detection of gastric cancer and its histological type, and to investigate their correlations with clinical data.

## Methods

### Patients

Ethical approval was obtained from the institutional ethics review board, but the requirement of informed consent was waived due to the retrospective nature of the study. A total of 153 patients with gastric cancer and 45 patients with preoperative endoscopic biopsy-diagnosed benign gastric wall lesions (30 gastric inflammation and 15 normal gastric wall) who underwent spectral CT scans and surgical intervention were retrospectively enrolled from June 2013 to June 2016. Considering that the conditions of gastric wall beyond the sampling site are not accessible, the patients with gastric inflammation and normal gastric wall were grouped together. The exclusion criteria included pathologically-confirmed non-adenocarcinomas, history of preoperative therapy (such as radiotherapy and chemotherapy), severe artifacts on CT images, non-measurable lesions and incomplete clinical data. All the included patients completed the entire CT exam, and their gastric cavities were well distended on cross-sectional CT images without artifacts, and the gastric adenocarcinomas were clearly distinguished from normal gastric wall. Clinical data of patients, such as age, gender tumor thickness, tumor location and tumor differentiation were also documented.

### CT scan protocol

After fasting for 8 h, patients were asked to consume 1000 mL of warm water and then injected with 20 mg of scopolamine (Specifications: 10 mg/mL; Hangzhou Minsheng Pharmaceutical Group Co., Ltd. Hangzhou, China) 10 min prior to examination. Patients were placed in the supine position, and scanned on GE Discovery CT750 HD scanner (GE Healthcare, Milwaukee, WI, USA) with gemstone spectral imaging (GSI) mode. Dual-energy CT images were acquired using a single x-ray source switches rapidly between 80 kVp and 140 kVp at less than 5 millisecond speed. The other acquisition parameters were as follows: 5 mm slice thickness, 40 mm detector coverage, 0.984 helical pitch, 630 mA tube current, 0.6 s rotation time, 512 × 512 matrix, and 40 × 40 cm field of view. AP and PP contrast-enhanced CT scans were performed with 40 and 70 s delays, respectively, after intravenous injection of 85–110 mL (1.5 mL per kg of body weight) iodinated contrast material (Ultravist 370, Bayer Schering Pharma, Berlin, Germany) at a rate of 3.0 ml/s through pump injector (Ulrich REF XD 2060-Touch, Ulrich Medical, Ulm, Germany). Contrast-enhanced CT images were reconstructed by using a standard kernel and 2.5 mm section thickness. The value of CT dose index volume (CTDIvol) for dual energy spectral mode in the abdomen was 23.84 mGy.

### Image analysis

All data were transferred to GE AW 4.6 workstation (GE Healthcare, Milwaukee, WI, USA), and interpreted by two radiologists with 6 and 10 years of experience in gastrointestinal radiology. Data analysis was carried out independently using GSI Viewer software (GE Healthcare, Milwaukee, WI, USA) with a standard soft-tissue window (WL 40 and WW 400). Regions of interest (ROI) were drawn on the solid part of the tumor (about two-thirds of the area), with the exclusion of peripheral fat, visible vessel, calcification and cystic/necrotic areas. A circular ROI was placed into the aortic arch within the same CT slice, after the exclusion of calcified atherosclerotic plaque. Subsequently, the thickness of tumour was measured and recorded. In order to reduce the individual variation between patients, IC value was normalized by dividing the IC of lesion to that of aorta (nIC=IClesion/ICaorta) [12]. All IC values were repeatedly measured three times, and the average value was then calculated. Similarly, ROI of the three gastric regions (fundus, body and antrum) was measured for three times, and their average values were calculated.

### Statistical analysis

All statistical analyses were performed with MedCalc v.9.2.0.0 (Frank Schoonjans, Broekstraat 52,B-9030 Mariakerke, Belgium). *P* values of less than 0.05 were considered statistically significant. Interobserver agreement for IC and nIC values was evaluated using intraclass correlation coefficient (ICC), which classified as poor (< 0.40), fair (0.40–0.59), good (0.60–0.74), or excellent (0.75–1.00). The values of IC and nIC at both AP and PP were expressed as median (P25, P75). Mann-Whitney U test was used to compare the IC values between cancer and benign gastric wall group, as well as the IC values among age, gender, tumor thickness and histological types. Kruskal-Wallis H test was used to compare the differences of IC and nIC values between different tumour sites, including funtus, body and antrum. Furthermore, ROC curves were used to evaluate the diagnostic values of IC and nIC in discriminating gastric cancer and its histological type.

## Results

### Clinical data

**Fig. 1:**
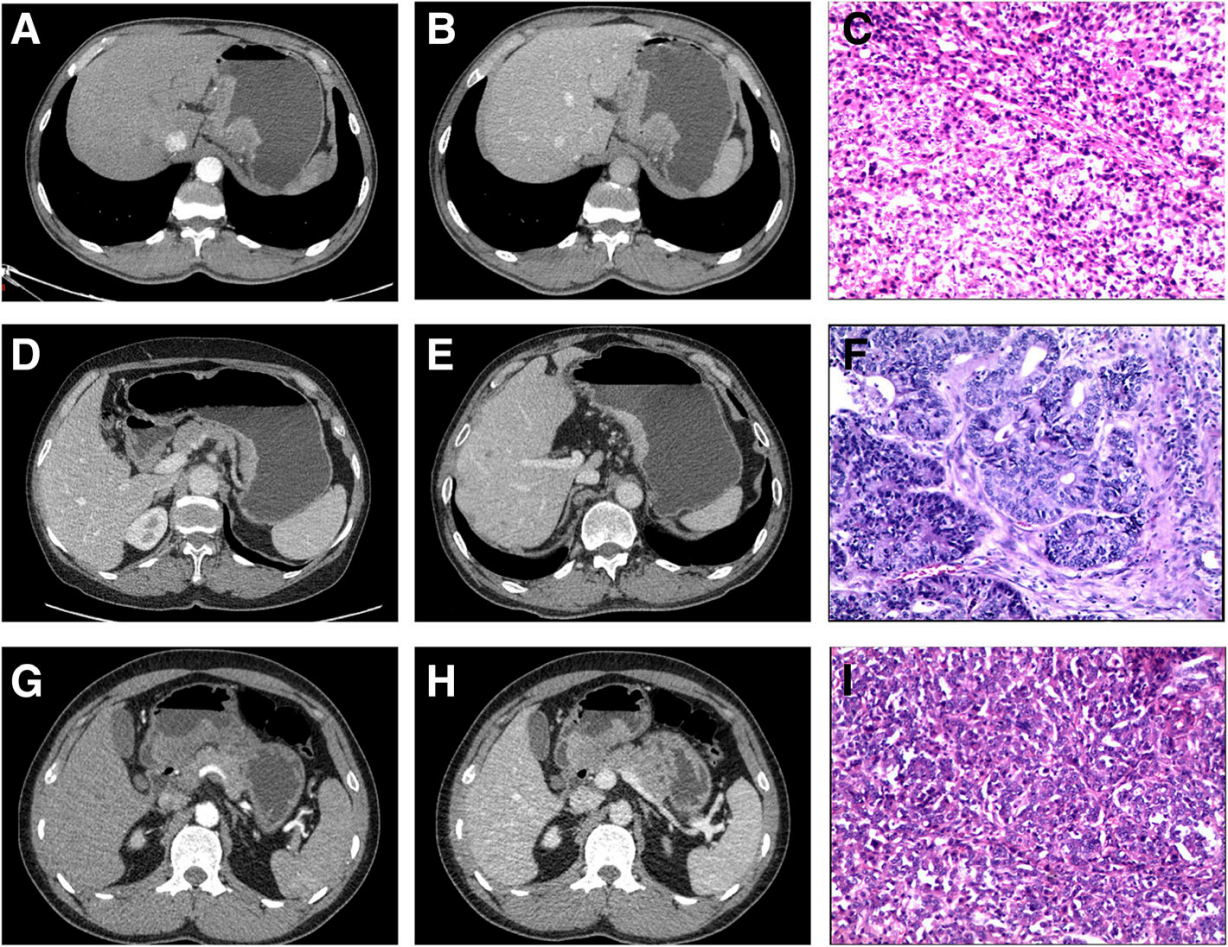
Spectral CT images of patients with poorly and moderately differentiated gastric adenocarcinomas at different sites. **a** AP image shows moderate enhancement of fundal wall thickening in a 67-year-old male patient. The maximum thickness is 17.89 mm, and ICAP is 16.78 (100 μg/ml). **b** PP image demonstrates a ICPP value of 27.19 (100 μg/ml) in the same patient. c Photomicrograph of histological specimen indicates a poorly differentiated adenocarcinoma [hematoxylin and eosin (H&E) stain; original magnification × 100]. **d** AP image shows irregular wall thickening of the gastric body in a 41-year-old female patient. The maximum thickness is 14.24 mm, and ICAP is 14.46 (100 μg/ml). **e** PP image demonstrates a ICPP value of 24.57 (100 μg/ml) in the same patient. **f** Photomicrograph of histological specimen indicates a poorly differentiated adenocarcinoma. (H&E stain; original magnification × 100). **g** AP image shows antrum wall thickening with surface ulcers in a 56-year-old male patient. The maximum thickness is 20.32 mm and ICAP is 11.79 (100 μg/ml). **h** PP image demonstrates a ICP*P* value of 20.19 (100 μg/ml) in the same patient. **i** Photomicrograph of histological specimen indicates a moderately differentiated adenocarcinoma (H&E stain; original magnification × 100)

A total of 87 (57%) out of 153 gastric cancer patients and 36 (80%) out of 45 patients with benign gastric wall lesions were ultimately included in the study. Among the excluded patients, 18 of them were diagnosed as non-adenocarcinoma by surgical pathology, 12 patients with poor image quality (severe artifacts) evaluated by two radiologists in consensus, and 8 patients received preoperative therapy. A further 9 patients with benign gastric wall lesions and 28 gastric cancer patients were excluded from analysis due to the non-measurable lesions on enhanced CT images. Overall, clinical data of 87 gastric adenocarcinoma patients and 36 patients with benign gastric wall lesions were used for final analyses. The clinical characteristics of all included patients are summarized in Table 1, while the images of gastric adenocarcinoma patients at different sites are shown in Fig. 1.

**Table 1:** Clinical characteristics of 87 patients with gastric adenocarcinomas and 36 patients with benign gastric wall lesions

Characteristics	Statistics(mean, range)
*Cancer peoples*	87
Age (years)	55 (29–74)
Gender(M/F)	60/27
Tumor thickness (cm)	3.1 (1.0–8.9)
Tumor site	
Fundus	29 (33.3%)
Body	28 (32.3%)
Antrum	27 (31%)
Whole stomach	3 (3.4%)
Histological differentiation degree	
Highly differentiated adenocarcinoma	4 (4.6%)
Moderately differentiated adenocarcinoma	40 (46.0%)
Poorly differentiated adenocarcinoma	43 (49.4%)
*Benign lesion peoples*	36
Age (years)	53 (32–72)
Gender(M/F)	22/14
Gastric wall	
Inflammation	25(69%)
Normal	11(31%)

Abbreviations:*M* Male, *F* Female

### Interobserver agreement

The interobserver agreement of IC measurement between two readers was ranked from good to excellent. In particular, the values of ICAP, ICPP, nICAP and nICPP in were 0.664, 0.755, 0.913 and 0.980, respectively, in gastric cancer group and 0.694, 0.713, 0.897, 0.910, respectively, in benign gastric wall lesions group. The mean difference between the two observers was used for further analysis.

### Comparison of IC and nIC values between benign gastric wall lesions and gastric cancer

**Fig. 2:**
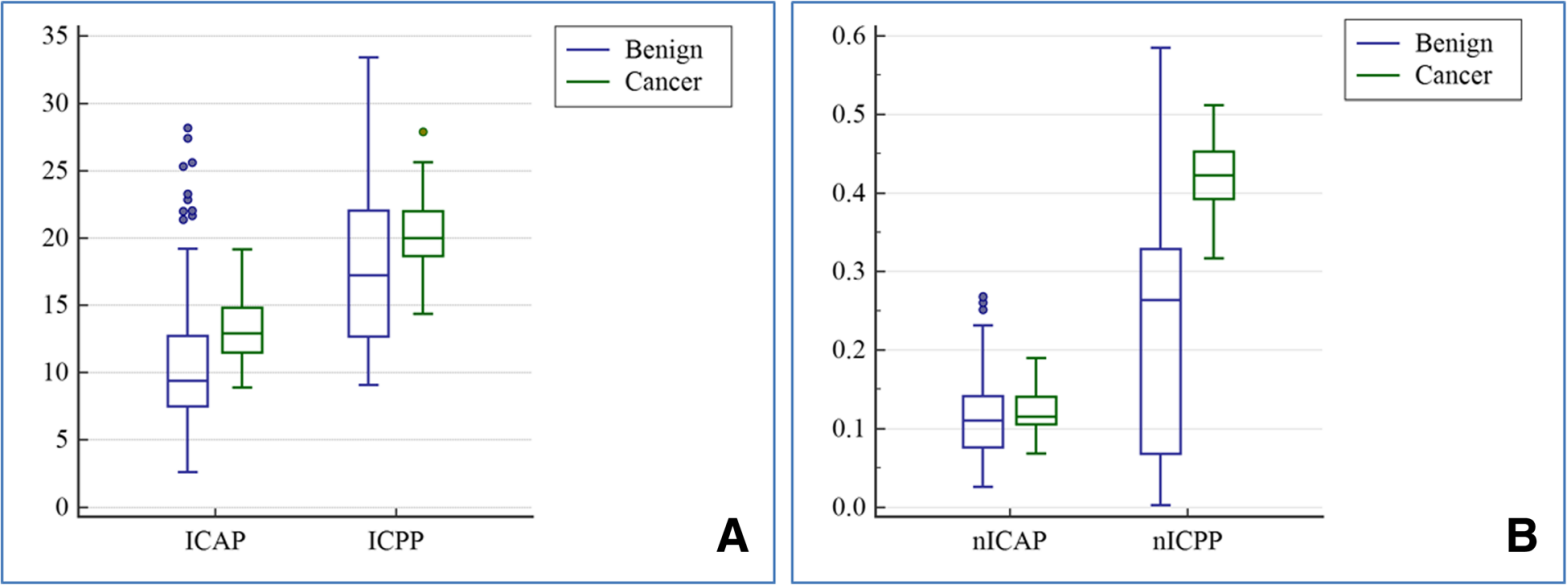
Differences in IC and nIC values between between gastric cancer and benign lesion groups. Box-and-whisker plots (box: 25, 75%; centreline: medium; whisker: min, max) reveal that (**a**) ICAP, ICPP, (**b**) nICAP and nICPP of gastric adenocarcinoma group were significantly higher than benign gastric wall group, with *P* < 0.0001, *P* = 0.0001, *P* = 0.0445 and *P* < 0.0001, respectively

As shown in Fig. 2 and Table 2, the values of ICAP, ICPP, nICAP and nICPP in benign gastric wall lesions group were 9.388 (7.497, 12.740) 100 μg/ml, 17.233 (14.448, 18.798) 100 μg/ml, 0.111 (0.076, 0.141) and 0.264 (0.068, 0.328), respectively. On the other hand, the values of ICAP, ICPP, nICAP and nICPP in gastric cancer group were 12.900 (11.508, 14.832) 100 μg/ml, 20.000 (18.623, 22.000) 100 μg/ml, 0.115 (0.105, 0.141) and 0.423 (0.392, 0.453), respectively. Notably, these values were significantly higher in gastric cancer group than those in benign gastric wall lesions group [*Z* = 5.870 (ICAP), 3.894 (ICPP), 2.009 (nICAP) and 10.137 (nICPP); *P* < 0.005].

**Table 2:** Comparison of IC and nIC values between gastric cancer and benign lesion groups

Group	Number	ICAP(100 μg/ml)	ICPP(100 μg/ml)	nICAP	nICPP
Benign	108	9.388 (7.497, 12.740)	17.233 (14.448, 18.798)	0.111 (0.076, 0.141)	0.264 (0.068, 0.328)
Cancer	87	12.900 (11.508, 14.832)	20.000 (18.623, 22.000)	0.115 (0.105, 0.141)	0.423 (0.392, 0.453)
*Z* value		5.870	3.894	2.009	10.137
*P* value		< 0.0001	=0.0001	=0.0445	< 0.0001

Abbreviations: *ICAP* arterial phase iodine concentration, *ICPP* portal venous phase iodine concentration, *nICAP* normalized arterial phase iodine concentration, *nICPP* normalized portal venous phase iodine concentration

### Comparison of IC and nIC values between gender, age, thickness, location and histological types of gastric adenocarcinoma

**Fig. 3:**
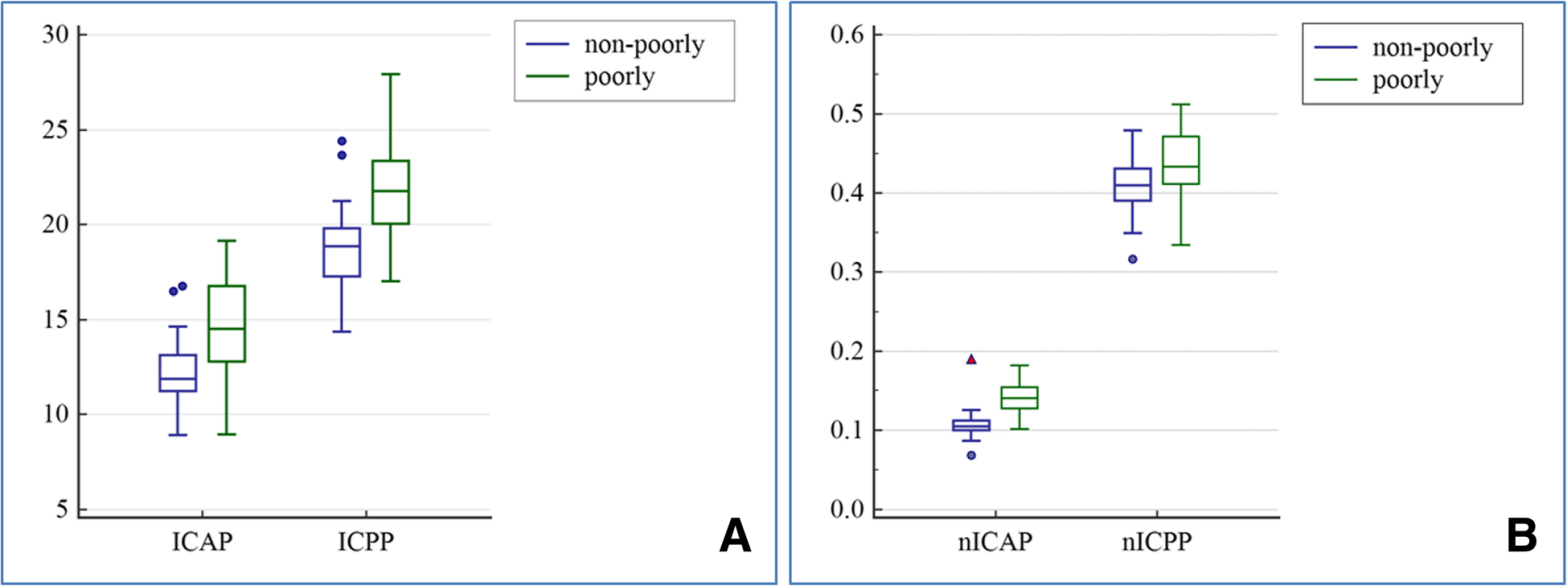
Differences in IC and nIC values between poorly and well-differentiated gastric adenocarcinoma groups. Box-and-whisker plots (box: 25, 75%; centreline: medium; whisker: min, max) indicate that (**a**) ICAP, ICPP, (**b**) nICAP and nICPP of poorly differentiated gastric adenocarcinoma group were significantly higher than well-differentiated gastric adenocarcinoma group, with *P* < 0.0001, *P* < 0.0001, *P* < 0.0001 and *P* = 0.0032, respectively

As compared to female patients, the values of ICAP, ICPP, nICAP and nICPP were slightly higher in male patients (Table 3). However, none of these differences were statistically significant [*Z* = 0.922 (ICAP), 1.372 (ICPP), 1.636 (nICAP) and 1.449 (nICPP); *P* >  0.05]. All gastric cancer patients were divided into young (≤55 years old) and older (> 55 years old) groups according to the mean age of 55 years old. The values of ICAP, ICPP, nICAP and nICPP in young group were slightly higher than those in older group, but the differences were not statistically significant (*Z* = 0.613, 1.066, 1.935 and 0.583, respectively; *P* >  0.05). In addition, the patients were divided into small (≤3.1 cm) and large (> 3.1 cm) tumor groups according to the mean thickness of 3.1 cm. The values of ICAP, ICPP, nICAP and nICPP in small tumor group were slightly higher than those in large tumor group, but not significantly different (*Z* = 1.083, 0.706, 0.103 and 1.272, respectively; *P* >  0.05). Similarly, the values of ICAP, ICPP, nICAP and nICPP were not significantly different among the three tumor location groups (*H* values = 0.205, 4.221, 1.859 and 4.836, respectively; *P* >  0.05). Furthermore, the moderately and well-differentiated gastric adenocarcinoma groups were combined into a single group, due to the limited numbers of cases in well-differentiated tumor group (*n* = 4). As shown in Figure 3, the values of ICAP, ICPP, nICAP and nICPP were significantly higher in poorly differentiated group than those in well-differentiated group (*Z* = 4.118, 5.637, 6.729 and 2.950, respectively; *P* < 0.05).

**Table 3:** Comparison of IC and nIC values between gender, age, location, thickness and histological types of gastric adenocarcinoma

Clinical features	Number of cases	ICAP (100 μg/ml)	ICPP (100 μg/ml)	nICAP	nICPP
*Gender*					
Male	60	13.018 (11.511, 15,780)	20.464 (18.843, 22.570)	0.125 (0.108, 0.147)	0.431 (0.398, 0.462)
Female	27	12.893 (11.456, 14.385)	19.480 (18.635, 21.485)	0.117 (0.105, 0.136)	0.412 (0.389, 0.443)
*Z* value		0.922	1.372	1.636	1.449
*P* value		> 0.05	> 0.05	> 0.05	> 0.05
*Age*					
Young(≤55 y)	41	13.010 (11.483, 16.675)	20.460 (18.868, 22.570)	0.138 (0.108, 0.153)	0.430 (0.400, 0.455)
Older (> 55 y)	46	13.009 (11.450, 14.440)	19.480 (18.780, 22.000)	0.119 (0.107, 0.136)	0.421 (0.389, 0.463)
*Z* value		0.613	1.066	1.935	0.583
*P* value		> 0.05	> 0.05	> 0.05	> 0.05
*Sites*					
Fundus	29	12.900 (11.543, 16.675)	20.010 (18.742, 22.240)	0.119 (0.106, 0.147)	0.423 (0.394, 0.435)
Body	28	13.672 (11.465, 15.787)	21.835 (19.815, 23.040)	0.140 (0.115, 0.151)	0.642 (0.404. 0.486)
Antrum	27	13.674 (11.930, 15.735)	20.200 (19.253, 22.108)	0.127 (0.111,0.141)	0.430 (0.399, 0.472)
*H* value		0.205	4.221	1.859	4.836
*P* value		> 0.05	> 0.05	> 0.05	> 0.05
*Thickness*					
Small (≤3.1 cm)	38	13.205 (11.890, 15.780)	20.332 (19.022, 22.105)	0.128 (0.109, 0.142)	0.433 (0.393, 0.468)
Large (> 3.1 cm)	49	12.780 (11.111, 14.532)	19.815 (18.783, 22.570)	0.119 (0.107, 0.147)	0.417 (0.396, 0.433)
*Z* value		1.083	0.706	0.103	1.272
*P* value		> 0.05	> 0.05	> 0.05	> 0.05
*Differentiation*					
Poorly	43	14.530 (13.168, 15.670)	21.780 (20.030, 23.348)	0.141 (0.127, 0.155)	0.433 (0.411, 0.472)
Well-differentiated	44	11.880 (11.240, 13.120)	18.855 (17.270, 19.800)	0.106 (0.100, 0.113)	0.410 (0.391, 0.431)
*Z* value		4.118	5.637	6.729	2.950
*P* value		< 0.0001	< 0.0001	< 0.0001	=0.0032

Abbreviations: *ICAP* arterial phase iodine concentration, *ICPP* portal venous phase iodine concentration, *nICAP* normalized arterial phase iodine concentration, *nICPP* normalized portal venous phase iodine concentration

### Diagnostic accuracy of IC values in detecting gastric cancer

**Fig. 4:**
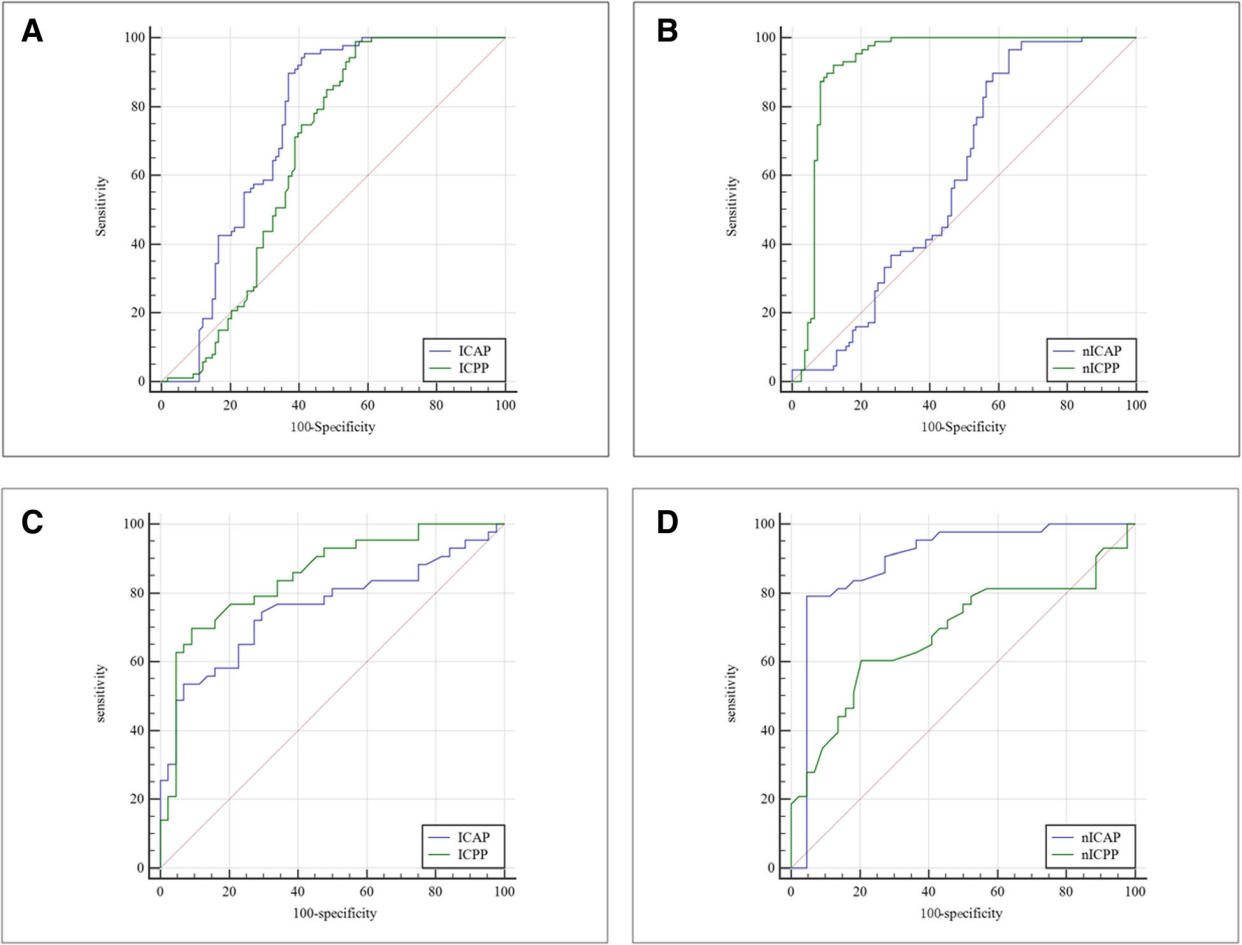
ROC curves for ICAP, ICPP, nICAP and nICPP. The ROC curves of (**a**) ICAP, ICPP, (**b**) nICAP and nICPP between gastric cancer and benign gastric wall lesions groups. nICPP has the highest AUC value, followed by ICAP, ICPP and nICAP. The ROC curves of (**c**) ICAP, ICPP, (**d**) nICAP and nICPP between poorly and well-differentiated gastric adenocarcinoma groups. nICAP has the highest AUC value, followed by ICPP, ICAP and nICPP

The AUC values of ICAP, nICAP, ICPP and nICPP for the gastric cancer detection were 0.745, 0.584, 0.662 and 0.923, respectively (Fig. 4 and Table 4). Of note, nICPP demonstrated the greatest ability in discriminating gastric cancer. Besides, the optimal cut-off values of ICAP, nICAP, ICPP and nICPP were 10.343, 0.089, 14.913 and 0.364, respectively. The sensitivities of ICAP, nICAP, ICPP and nICPP were 95.40, 96.55, 98.85 and 91.95%, respectively; while the specificities were 58.33, 37.04, 43.52 and 87.96%, respectively.

**Table 4:** The ROC curves of IC and nIC values

ROC	ICAP	ICPP	nICAP	nICPP
Cancer				
AUC	0.745	0.662	0.584	0.923
sensitivity	95.40%	98.85%	96.55%	91.95%
specificity	58.33%	43.52%	37.04%	87.96%
Optimum cutoff values	10.343	14.913	0.089	0.364
Youden index	0.537	0.424	0.336	0.799
Poorly differentiated				
AUC	0.756	0.851	0. 919	0.684
sensitivity	53.49%	69.77%	79.07%	60.47%
specificity	93.18%	90.91%	97.73%	79.55%
Optimum cutoff values	14.460	20.461	0.125	0.431
Youden index	0.467	0.607	0.768	0.400

Abbreviations: *ROC* Receiver operating characteristic curves, *AUC* area under the curve, *ICAP* arterial phase iodine concentration, *ICPP* portal venous phase iodine concentration, *nICAP* normalized arterial phase iodine concentration, *nICPP* normalized portal venous phase iodine concentration

### Diagnostic accuracy of IC values in discriminating the histological types of gastric adenocarcinoma

The AUC values of ICAP, nICAP, ICPP, and nICPP in discriminating poorly and well-differentiated gastric adenocarcinoma were 0.756, 0.919, 0.851 and 0.684, respectively (Fig. 4 and Table 4). Of note, nICAP demonstrated the greatest ability in discriminating the histological types of gastric adenocarcinoma, whereas nICPP showed the lowest. The optimal cut-off values of ICAP, nICAP, ICPP and nICPP were 14.460, 0.125, 20.461 and 0.431, respectively. The sensitivities of ICAP, nICAP, ICPP, and nICPP were 53.49, 79.07, 69.77 and 60.47%, respectively; while the specificities were 93.18, 97.73, 90.91 and 79.55%, respectively.

## Discussion

Spectral CT extends the capabilities of conventional CT, which uses a rapid kilovoltage switching technique to acquire monochromatic images of tissues, in a similar way to those obtained from a single X-ray source [18–20]. Subsequent elemental decomposition analysis can be performed to obtain iodinated contrast attenuation map, thereby allowing iodine density to be calculated [21, 22]. As a result, this can assist the radiologists to address diagnostic errors. Hence, the present study investigated the role of quantitative spectral CT parameters for the discrimination of gastric cancer and its histological types, and examined their correlations with clinical features. The major findings of this study were as follow: (1) IC values in gastric cancer were higher than benign gastric wall lesions, in which nICPP demonstrated the greatest diagnostic efficacy; (2) IC values in poorly differentiated gastric adenocarcinoma were higher than in well-differentiated caners, in which nICAP showed the highest diagnostic efficacy; and (3) IC values were not significantly different between age, gender, tumor thickness and tumor location.

Iodine concentration reflects the vessel density and the blood volume in different tissue regions during a contrast-enhanced CT scan. Tang et al. [15] reported a high consistency between spectral CT-measured IC and actual IC, and thus it is a useful parameter to indicate the physiological function. The growth and progression of solid tumors depend upon the formation of new blood vessels, which is different from normal tissues or benign lesions. Several studies have reported that CT imaging is useful for distinguishing small hepatocellular carcinoma from other hepatic lesions [23, 24], small intrahepatic mass-forming cholangiocarcinoma from small liver abscess [25], malignant from benign pulmonary nodules [26], and gastric cancer from benign gastric mucosal lesions [11]. Indeed, the quantitative IC measurement is significantly higher in cancerous lesions compared to benign lesions, and its accuracy is greater than that of conventional CT. In addition, Liu et al. examined the patients with papillary thyroid cancer, and their results suggested that nIC measured during AP and PP are significantly higher in metastatic lymph nodes as compared to benign lesions [27]. Taken together, our results are consistent with the aforementioned studies, in which the values of IC are higher in cancer than in benign lesions, due to the increased angiogenesis during tumor development, leading to their enhancement in CT scan [28].

The degree of tumor differentiation is a predictive and prognosis biomarker for patients with gastric cancer [29], which can be distinguished quantitatively by dual-energy spectral CT (DESCT). Pan et al. [12] evaluated the clinical usefulness of DESCT in the classification and staging of gastric cancer. Their findings indicated that monochromatic images obtained from DESCT can be used to improve the accuracy of preoperative staging, and quantitative IC measurement is helpful in distinguishing the poorly and well-differentiated gastric carcinoma, as well as the metastatic and non-metastatic lymph nodes. A similar pattern of results was obtained, where IC and nIC values were significantly lower in well-differentiated gastric cancer compared to poorly differentiated ones, which can be explained by the differences in tumor angiogenesis. Du et al. [30] have suggested that vascular endothelial growth factor (VEGF) expression and microvessel density (MVD) are closely correlated with histological degree, in which their levels are reduced in early stage gastric carcinoma compared to progressive carcinoma. Chang et al. [31] have reported that MVD is significantly associated with poorly differentiated gastric adenocarcinoma. Moreover, Hu et al. [32] demonstrated that the nIC value of three-phase enhanced CT scan is positively correlated with MVD. Additionally, Chen et al. reported that poorly differentiated gastric cancer exhibited higher MVD and nIC value, and a positive correlation between them [33]. A CT perfusion study on gastric cancer has revealed that the lower the degree of tumor differentiation, the higher the permeability surface area [34]. These findings indicate that poorly differentiated tumors may increase vasopermeability and immature endothelial cells, thereby explaining the high values of IC and MVD in gastric patients with poorly differentiated adenocarcinoma.

nIC can minimize the effects of individual variability, such as contrast dose, injection rate and individual differences in circulation, and thereby it is more efficacy than IC. Both nICAP and nICPP were found to be significantly different between gastric cancer group and benign gastric wall lesions group, as well as between poorly differentiated group and well-differentiated group. In particular, nICAP demonstrated a higher efficiency in the diagnosis of poorly differentiated gastric adenocarcinoma, with AUC, sensitivity and specificity of 0.896, 79.07 and 95.45%, respectively. Meanwhile, nICPP showed a higher efficacy for the detection of gastric cancer, with AUC, sensitivity and specificity of 0.923, 91.95 and 87.96%, respectively. These results can be partly explained by different functional roles of nICAP and nICPP. nICAP mainly reflects the capillary density and the blood supply of gastric carcinomas, while nICPP may indicate the flow of blood supply and the retention of contrast agent in intrasvasular and extravascular space following AP. It is noticeable that venous phase enhancement is more prominent in gastric cancer, suggesting that PP enhancement characteristics are more useful for the detection of gastric cancer. Besides, the reason why PP is less effective than AP in distinguishing histological types may be due to the influence of blood flow. In addition, nICPP has been reported to exert a high sensitivity in differentiating malignant gastric mucosal lesions from normal gastric mucosa [11]. Furthermore, studies on the diagnostic efficacy of tumor differentiation degree are inconsistent [35, 36], which may be due to the different biological behaviors of tumors and degrees of differentiation. A previous study has found that the values of IC and nIC of poorly differentiated adenocarcinoma are not significantly different compared to those of moderately and well-differentiated adenocarcinomas in AP [17]. These inconsistent results may be attributed to different patient populations and scan protocol. In that study, patients were subjected to triple-phase CT imaging, including AP at 25 s, which are different from ours. In sum, DESCT is more useful in evaluating gastric cancer with a delay AP scan protocol.

Apart from that, Karim et al. [37] reported that younger age is correlated with the histology grade of gastric cancer. However, the histology grade is not correlated with gender and tumor location [38]. In addition, Wang et al. [38] demonstrated that tumour size is a prognostic factor in patients with advanced gastric cancer. In the present study, no significant differences were found in the values of ICAP, ICPP, nICAP and nICPP between age, gender, tumor thickness and tumor location.

There are some unavoidable limitations in this preliminary study. First, DESCT scans were performed on the first-generation Discovery CT750 HD scanner with a fixed mA value of 600 mA. This yielded a CTDIvol of 23.84 mGy, which is considerably high in current clinical practise settings. With the introduction of the second-generation CT750 HD scanner, the radiation dose has been reduced to 30% in GSI mode, and further dose reduction is forthcoming. Second, this is a retrospective study, and the sample size was relatively small, especially the number of patients with well-differentiated adenocarcinoma was too low to to allow a statistical comparison with moderately and poorly differentiated ones. Third, only patients with gastric adenocarcinoma were enrolled in this study, patients with other histological types of gastric cancer were not taken into account. Moreover, it was difficult to obtain pathologic confirmation of the entire gastric wall in non-cancer patients, and the patients with gastric inflammation and normal gastric wall were grouped together. Finally, since DESCT is a relatively new technique for gastric cancer, it may hinder the adaptation process based on these preliminary results.

## Conclusions

In conclusion, this preliminary study compared the quantitative spectral CT parameters between benign lesion and gastric cancer groups, as well as different histological types of gastric adenocarcinoma. IC values can be used to accurately identify gastric cancer and quantitatively assess the degree of differentiation, without being affected by age, gender, tumor thickness and tumor location. These findings may improve the preoperative staging of gastric cancer, and lay the foundations for modern functional imaging in oncology. However, further studies with larger sample sizes are needed to draw a firm conclusion.

## Abbreviations

AP: Arterial phase
AUC: Area under the curve
CT: Computed tomography
CTDIvol: CT dose index volume
DEsCT: Dual-energy spectral CT
IC: Iodine concentration
ICC: Intraclass correlation coefficient
MD: Material decomposition
MVD: Microvessel density
nIC: Normalized iodine concentration
PP: Portal venous phase
ROC: Receiver-operating characteristics analysis
ROI: Regions-of-interest
SD: Standard deviation
VEGF: Vascular endothelial growth factor
